# Focus on the Beauty of Body: The Mediation Role of Body Appreciation Between Perfectionism and Body-Related Shame and Body-Related Guilt

**DOI:** 10.3389/fpsyg.2021.638641

**Published:** 2021-07-26

**Authors:** Zhang Liang, Shen Lingting, Cai Ying, Liu Xiaoyan, Zhang Yan, Ying Ronghua, Bi Dan, Tao Yanqiang

**Affiliations:** ^1^Student Mental Health Education Center, Northeast Agricultural University, Harbin, China; ^2^School of International Studies, Hangzhou Normal University, Hangzhou, China; ^3^Student Development Guidance Center, Student Affairs Office, Harbin Engineering University, Harbin, China; ^4^Beijing Key Laboratory of Applied Experimental Psychology, Faculty of Psychology, Beijing Normal University, Beijing, China; ^5^College of Humanities and Social Sciences, Harbin Engineering University, Harbin, China; ^6^School of Psychology, Nanjing Normal University, Nanjing, China; ^7^Yichun 1st high school, Yichun, China

**Keywords:** body appreciation, healthy perfectionism, unhealthy perfectionism, body-related shame, body-related guilt

## Abstract

**Objectives**: According to traditional views, perfectionists are prone to experience shame and guilt. As a relative part of negative body image, body appreciation reflects an appreciation attitude toward physical characteristics, functionality, and health, accepting and appreciating all parts and functions of the body, predicting body-related shame and guilt.

**Methods**: Therefore, body appreciation was examined for its potential mediating role in the relationship between two dimensions of perfectionism (e.g., healthy perfectionism and unhealthy perfectionism) and body-related shame and body-related guilt among 514 females.

**Results:** The results highlight that body appreciation partially mediated the relationship between perfectionism and body-related shame and body-related guilt. Implications for enhancing body appreciation among females between experiencing healthy or unhealthy perfectionism and body-related shame and body-related guilt feelings are discussed.

**Conclusions**: These findings underscore the importance of considering body appreciation in addressing perfectionism dimensions and body-related shame and body-related guilt. Research and clinical implications are also addressed.

## Introduction

Women generally have a negative body image, low self-confidence, negative avoidance of thoughts, and constant dissatisfaction with their appearance and weight (Frederick et al., 2016), which result in negative appearance evaluations (Frederick et al., 2007; Fiske et al., 2014). Moreover, this dissatisfaction may lead to physical anxiety (Cash et al., 2004), excessive exercise (White and Halliwell, 2010; Yager et al., 2017), unhealthy diet (Cash and Iii, 1997; Slevec and Tiggemann, 2011), and eating disorders (Stice and Shaw, 2002; Hoek and van Hoeken, 2003), which is not conducive to women’s physical and mental development.

As two essential aspects of negative body image in women, body-related shame and body-related guilt have received growing research attention (Bessenoff and Snow, 2006; Mensinger et al., 2018). Body-related shame is a self-conscious emotion that negatively evaluates their *global* self (Tangney, 1998). Body-related guilt is a negative self-conscious emotion that occurs when an individual has a negative evaluation of his/her *behavior* (Tangney, 1998). Shame and guilt have been associated with depression (Brunet et al., 2019), anxiety (Hedman et al., 2013) in the general adult population. Although body-related shame and body-related guilt are self-conscious emotions that often co-occur, they have been differentiated in prior scholarship along cognitive, affective, and motivational dimensions (Lewis, 1971; Tangney, 1992).

Furthermore, there is no professional scale of body-related shame and body-related guilt in China as far as we know. Hence, the main goal of this study is to successfully find the potential predictors that can influence adult women’s body-related shame and guilt, which could provide constructive suggestions to individuals who are dissatisfied with their body image in clinical consultation. Then, finally, we can promote a positive stance toward their body image.

### Perfectionism and Body-Related Shame and Body-Related Guilt

Hamachek (1978) conceptualized and operationalized perfectionism as multidimensional construct comprised of positive and negative dimensions. Positively, perfectionism is defined as a lifestyle that could reduce anxiety (Gol et al., 2013) or an accurate, comprehensive, correct, non-life-threatening solution to human problems (Fedewa et al., 2005). Negatively, perfectionism has been conceptualized as a pathology-causing personality trait (Frost et al., 1990; Hewitt and Flett, 1991) or an emotion with a lot of psychological barriers (Burns, 1980; Pacht, 1984).

In adsdition to the Hamachek’s theory, Flett and Hewitt (2002) classified perfectionism as adaptive and maladaptive. Adaptive perfectionism is an achievable high standard of organization and planning, which can feel self-satisfaction and a sense of accomplishment after the completion of the plan (Enns and Cox, 2002; Franco-Paredes et al., 2005). In contrast, maladaptive perfectionism is an unrealistic standard that accompanies critical evaluation, such as questioning one’s behavior, being overly concerned with mistakes, and attributing failure to mistakes (Boone et al., 2010). Frost et al. (1990) divided perfectionism into two aspects according to their own and their parents’ requirements and subdivided it into six dimensions to develop the Frost Multi-dimensional Perfectionism Scale (FMPS). Based on this, Stumpf and Parker (2000) divided it into healthy perfectionism and unhealthy perfectionism, corresponding to normal and neurotic perfectionism according to Hamachek’s theory (Hamachek, 1978), respectively. And then, Hongfei (2007), a Chinese scholar, revised it and merged six dimensions into two dimensions under the Chinese cultural environment. In this study, the operational definition of perfectionism is adopted by Hongfei (2007), who supposed that perfectionism is the pursuit of no defects and the adoption of high standards for oneself, and divided perfectionism into two forms: healthy perfectionism (also named as adaptive perfectionists) and unhealthy perfectionism (also named as maladaptive perfectionists). Moreover, combined with the cluster analysis, we finally choose healthy perfectionism and unhealthy perfectionism.

As we know that when an individual perceives that his or her body is unwelcome, which could cause a negative emotion (Dalley et al., 2020). Based on the self-objectification theory, the internalization of feminine appearance ideals elicits body-related self-conscious emotions (i.e., body-related shame and body-related guilt, etc.), moreover increased emotional problems (Fredrickson and Roberts, 1997). A large body of research suggested a general link between perfectionism and body-related shame and guilt (Giancarlo et al., 2015; Piotrowski, 2019; Cella et al., 2020). Hamachek (1978) proposed that moderate perfectionism would have shame and guilt, while normal perfectionism might experience pride instead of shame and guilt. Fedewa et al. (2005) found a positive correlation between negative perfectionism and body-related shame and guilt. However, positive perfectionism is only negatively related to shame. Similarly, a study reported that both positive and negative perfectionists tend to feel guilty to varying degrees, and only negative perfectionists tend to feel more ashamed (Stoeber et al., 2007).

Cumulative evidence indicates that compared to unhealthy perfectionists, healthy perfectionists show higher levels of indicators of good adjustment (e.g., positive effect) and lower levels of indicators of maladjustment (e.g., negative effect; Stoeber and Otto, 2006). Consequently, it can be expected that healthy perfectionists experience more pride and less shame and guilt than unhealthy perfectionists, which will display the characteristics associated with the positive perfectionism but not those associated with the negative perfectionism. Specifically, perfectionism could be regarded as a compensation mechanism, which means people can apply this mechanism to deal with inferiority, inadequacy, and fear of rejection (Hewitt et al., 2003). In this line, Peterson (2003) suggested that presenting a perfect image and concealing perceived flaws and defects aim to avoid shame experiences and may emerge as a strategy to achieve acceptance and fit in the group. Hence, the association between different forms of perfectionism and body-related shame and guilt needs to be explored furtherly.

### Body Appreciation as a Mediator

As a relative part of negative body image, positive body image gradually became the focus of scholars’ attention and was regarded as a method to improve negative body image (Wood-Barcalow et al., 2010; Webb et al., 2015). Positive body image is complete love and respect for the body, making people appreciate their bodies’ unique beauty and function, ignoring imperfections, internalizing positive information, and rejecting negative information (Wood-Barcalow et al., 2010). Considering that body appreciation is a part of a positive body image, reflecting an appreciation attitude toward physical characteristics, functionality, and health, which accepts and appreciates all parts and functions of the body (Avalos et al., 2005). However, fewer studies investigating potential mediators between perfectionism and body-related shame and body-related guilt emphasize the role of body appreciation.

The link between body perfectionism and body-related shame and body-related guilt has been understated. A study recruited 181 female college students from Midwest University and showed that body appreciation was negatively correlated with body-related shame, and body appreciation could significantly negatively predict body-related shame (Avalos et al., 2005). Pidgeon tested 70 female college students and divided them into religious, health, and control groups. Results showed that reducing body-related guilt and significantly improved body appreciation in the religious group (Pidgeon and Appleby, 2014). Studies recently demonstrated that the body image-related perfectionism plays a central role in the relationship between shame and eating pathology (Ferreira et al., 2015; Marta-Simões and Ferreira, 2016). More interesting, Iannantuono and Tylka (2012) found that perfectionism can significantly predict body appreciation, which maladaptive perfectionism can significantly negatively predict body appreciation, while adaptive perfectionism can significantly positively predict body appreciation.

In summary, body appreciation is a common underlying issue related to perfectionism, body-related shame, and body-related guilt. However, given the dimensionality of perfectionism, it is not clear how body appreciation and body-related shame, and body-related guilt relate to healthy and unhealthy perfectionism. Furthermore, it is unclear what role body appreciation plays in the link between perfectionism and body-related shame and body-related guilt.

### Aim of the Present Study

To sum up, the perfectionism literature (Avalos et al., 2005; Iannantuono and Tylka, 2012) suggests that perfectionism dimensions are linked directly or indirectly with body-related shame and body-related guilt *via* its association with body appreciation. However, previous studies have not been as body appreciation as predictors for studying body-related shame and body-related guilt. Therefore, we examined the mediating effects of body appreciation on the relationship between the perfectionism dimension (i.e., healthy perfectionism and unhealthy perfectionism) and body-related shame and body-related guilt.

## Materials and Methods

### Participants

Ethical approval was gained from the first author’s University Ethics Committee. The participants were 544 female students recruited from Harbin, a city in China. All participants ranging in age from 18 to 30 (*Mean* = 21.58, *SD* = 2.8). Their major included Science (50.4%) and Art (49.6%). Additionally, 29.0% from rural areas and 71.0% from urban areas. Of them, 59.9% are undergraduates and 40.1% are master’s students.

### Measures

#### The Chinese Frost Multidimensional Perfectionism Scale

The Chinese Frost Multidimensional Perfectionism Scale (Fei and Xu, 2006) comprises 27 items answered on a 5-point Likert scale (1 = *Strongly disagree* to 5 = *Strongly agree*). The questionnaire assesses five factors: personal standards (PS), parental expectations (PE), doubts about actions (DA), concern over mistakes (CM), and organization (O).

According to Stumpf and Parker's (2000) research, the scale is divided into healthy perfectionism and unhealthy perfectionism. Healthy perfectionism included PS and O; unhealthy perfectionism included PE, DA, and CM. In the present study, the Cronbach alpha coefficients were 0.81 for healthy perfectionism and 0.81 for unhealthy perfectionism.

#### The Body Appreciation Scale-2

The Body Appreciation Scale-2 (Tylka and Wood-Barcalow, 2015) comprises 10 items rated on a 5-point Likert scale format (1 = *Strongly disagree* to 5 = *Strongly agree*) and has been translated into Chinese (Swami et al., 2016). Item scores are averaged; higher scores imply greater body appreciation. The total BAS-2 scale with a Cronbach alpha coefficient of 0.84 was used in the present study.

#### Body-Focused Shame and Guilt Scale

The Body-Focused Shame and Guilt Scale (Weingarden et al., 2016) is a self-report questionnaire designed to measure shame, guilt, or externalization of the blame with body dysmorphic disorder (BDD). The BF-SGS is a scenario-based measure modeled after the TOSCA, which consists of 13 scenarios. Participant scores are rated from 1 (Not likely) to 5 (Very likely), such that higher total scores indicate greater body-related shame, body-related guilt, and externalization of blame.

We invited two Chinese-English bilingual scholars to translate these questionnaires from Chinese back into English to increase precision. The standard back-translation technique (Brislin, 1970) was employed to translate the BF-SGS, which the first author, a native Chinese speaker, used. Following that, the back-translated and the original English versions of these questionnaires were compared and corrected minorly. Reliability was also supported in the present study for body-related shame (Cronbach’s alpha = 0.88) and body-related guilt (Cronbach’s alpha = 0.82).

### Procedure and Analytic Plan

Ethical approval was gained from the first author’s University Ethics Committee. All participants completed questionnaires in a quiet classroom after obtaining informed consent, and they were free to withdraw from the study at any time. This procedure took about 25 min to complete all the questionnaires.

Before formal analysis, we deleted the outliers (*n* = 30). Then, we performed the independent T-test and one-way ANOVA analysis to test whether there is a significant difference between the dependent variables and the demographic variables. Finally, correlation analysis was to test the relationship between research variables. We conducted descriptive statistics using the package of compareGroups (Subirana et al., 2020), Pearson’s *r* correlations using the corrplot package (Wei and Simko, 2017).

Moreover, according to Baron and Kenny (1986), statistically significant correlations between predictors, possible mediators, and dependent variables are necessary for testing the mediation models. Hayes (2013) suggested that the correlation between X and Y is neither sufficient nor necessary to claim that X affects Y. Both paths, between X and mediator (the path a) and between the mediator and Y (the path b), are expected to be statistically significant.

Nonetheless, we followed Baron and Kenny’s assumption by initially estimating the correlation coefficients among all variables. Subsequently, following Hayes’ recommendation, we completed a series of multiple parallel mediation analyses (processR package, model 4) with the healthy perfectionism and unhealthy perfectionism as predictors (in different models), body appreciation as mediators, and body-related shame and body-related guilt as dependent variables, respectively. Finally, we used the bootstrapping method with a bootstrap value of 5,000 to examine indirect effects (Hayes, 2013). The 95% confidence intervals (CI) assigned the effect as significant if they did not include zero. Finally, we conducted mediation analysis using the package of processR.

## Results

### Descriptive Statistics and Correlations

Demographic characteristics of the samples are displayed in Table 1. As expected, there is no significant difference between the dependent variables and the demographic variables. Therefore, there is no need to include demographic variables in the mediation analysis.

**Table 1:** Test of the demographic variable on the dependent variable.

		Body shame (*n* = 514)	Body guilty (*n* = 514)
Background			
Marriage	Married [48 (9.34%)]	*F* (2,511) = 1.257, *p* > 0.05	*F* (2,511) = 0.372, *p* > 0.05
	Unmarried, have lovers [183 (35.6%)]		
	Unmarried, no lovers [283 (55.1%)]		
Birthplace	Rural [147 (28.6%)]	*F* (2,511) = 2.260, *p* > 0.05	*F* (2,511) = 2.578, *p* > 0.05
	Town [158 (30.7%)]		
	City [209 (40.7%)]		
Profession	Science [263 (51.2%)]	*t* (512) = −1.71, *p* > 0.05, *d* [95% CI] = 0.15 [−0.32, 0.02]	*t* (512) = −0.97, *p* > 0.05, *d* [95% CI] = 0.09 [−0.26, 0.09]
	Liberal [251 (48.8%)]		

**Figure 1:**
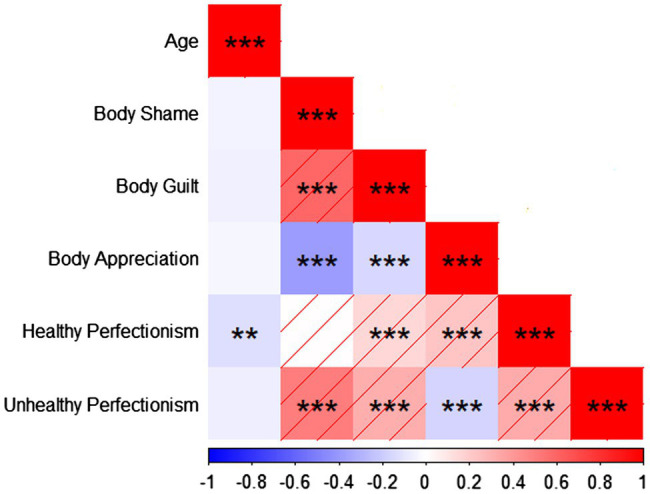
The red shade indicates a positive correlation. The red color represents a significantly positive correlation; the blue color represents a significantly negative correlation. ^**^p < 0.01; ^***^p < 0.001. The horizontal axis represents the correlation coefficient in the ± 1.

Figure 1 shows that the correlational analysis indicated positive associations between body-related shame and unhealthy perfectionism (*r* = 0.505, *p* < 0.001). Moreover, body-related guilt showed a positive relation to healthy perfectionism (*r* = 0.151, *p* < 0.001) and unhealthy perfectionism (*r* = 0.319, *p* < 0.001). The results also detected the negative relationship between body appreciation and body-related shame (*r* = −0.381, *p* < 0.001) and body-related guilt (*r* = −0.157, *p* < 0.001). Additionally, we noticed a positive relationship between body appreciation and healthy perfectionism (*r* = 0.224, *p* < 0.001). In contrast, a negative relationship between body appreciation and unhealthy perfectionism (*r* = −0.167, *p* < 0.001).

### Mediation Analysis

**Figure 2:**
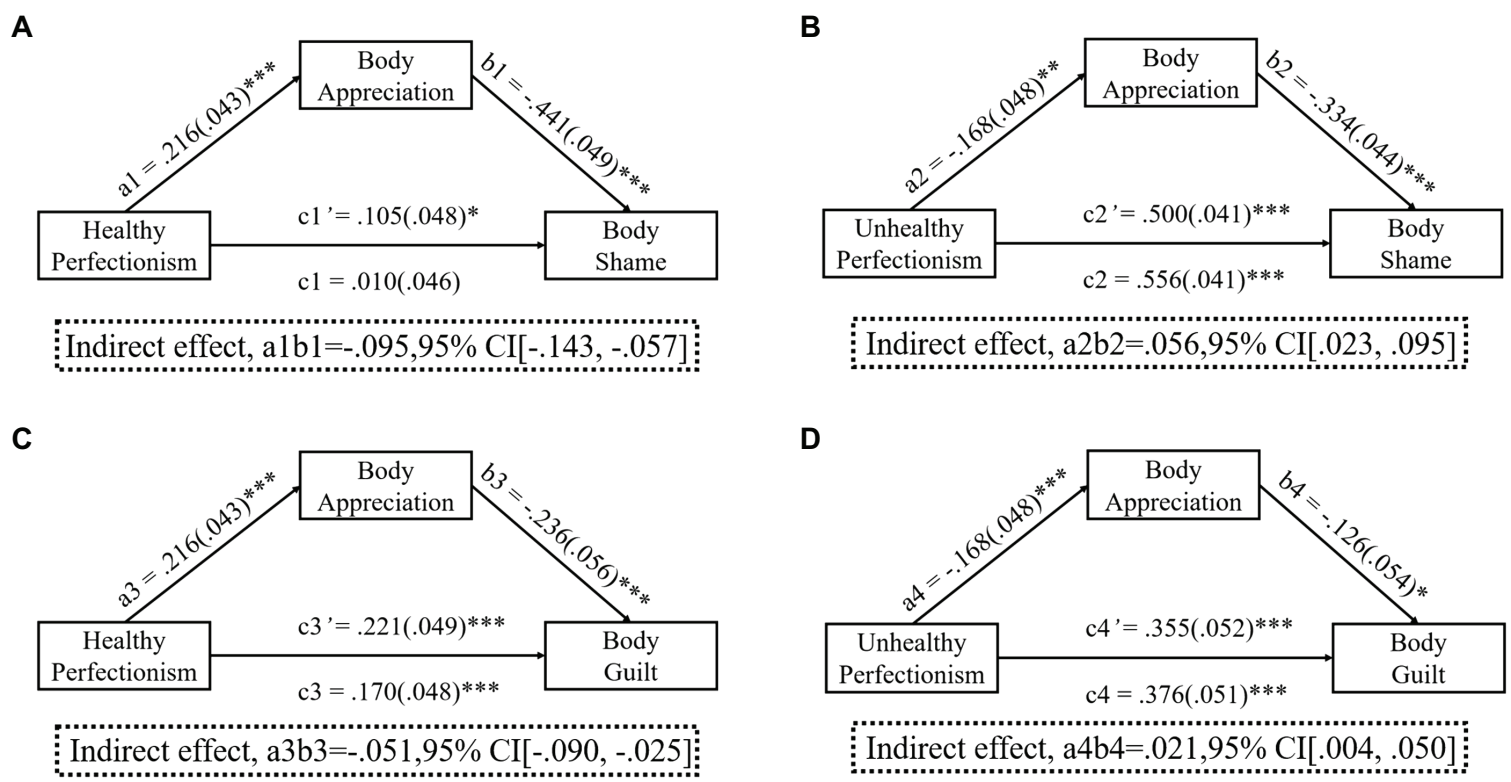
Results of mediating analysis. **(A)** The mediating effect of body appreciation in the relationship between healthy perfectionism and body-related shame. **(B)** The mediating effect of body appreciation in the relationship between unhealthy perfectionism and body-related shame. **(C)** The mediating effect of body appreciation in the relationship between healthy perfectionism and body-related guilt. **(D)** The mediating effect of body appreciation in the relationship between unhealthy perfectionism and body-related guilt. ^***^p < 001, ^**^p < 0.01, ^*^p < 0.05. Pathways represent unstandardized coefficients with SE in parentheses.

The relationship between healthy perfectionism and body-related shame (*c*1 = 0.010, *p* > 0.050) was mediated by body appreciation, as seen from the simple mediation analysis results is presented in Figure 2A. Specifically, the higher the scores on healthy perfectionism, the more body appreciation was felt (a1 = 0.216, *p* < 0.001), and subsequently, the more body appreciation felt, the lower body-related shame (*b*1 = −0.441, *p* < 0.001). A 95% CI based on 5,000 bootstrap samples indicated that the indirect effect (*a*1*b*1 = −0.095) was below zero (−0.143 to −0.057). Additionally, high healthy perfectionism was positively related to body-related shame (*c*1' = 0.105, *p* < 0.001), even after considering the indirect effect of body appreciation. Body appreciation partially mediated the relationship between healthy perfectionism and body-related shame.

Individuals with high unhealthy perfectionism felt body appreciation less often (*a*2 = −0.168, *p* < 0.010). The more body appreciation felt, the lower body-related shame experienced (*b*2 = −0.334, *p* < 0.001). A 95% CI based on 5,000 bootstrap samples indicated that the indirect effect (*a*1*b*1 = 0.056) was above zero (0.023–0.095). Furthermore, higher unhealthy perfectionism was accompanied by higher body-related shame (*c*2 = 0.556, *p* < 0.001), even when taking into account unhealthy perfectionism’s indirect effect through the mediator of body appreciation (*c*2' = 0.500, *p* < 0.001). The parameters of the indirect effect are displayed in Figure 2B. Body appreciation partially mediated the relationship between unhealthy perfectionism and body-related shame.

Figure 2C shows that the association between healthy perfectionism and body-related guilt was mediated by body appreciation (indirect effect; *a*3*b*3 = −0.051, CI 95% [−0.090, −0.025]). Body appreciation partially mediated the relationship between healthy perfectionism and body-related guilt. Figure 2D shows that the association between unhealthy perfectionism and body-related guilt was mediated by body appreciation (indirect effect; *a*4*b*4 = 0.021, CI 95% [0.004, 0.050]). Body appreciation partially mediated the relationship between unhealthy perfectionism and body-related guilt.

## Discussion

There is a growing body of literature on body-related shame and body-related guilt and their role in mental health. However, less is known about whether healthy or unhealthy perfectionism and mediating body appreciation affect body-related shame and body-related guilt. Consistent with our hypotheses, results indicated that healthy perfectionism positively predicted body appreciation. However, unhealthy perfectionism negatively predicted body appreciation, which is similarly consistent with the previous study (Iannantuono and Tylka, 2012). At the same time, body appreciation is a subjective positive attitude toward the body (Swami et al., 2016), which could be adjusted by personal self-willing. Healthy perfectionism has a positive strengthening effect on individual behavior, making things perfect by self-will and power. By contrast, unhealthy perfectionism has a negative reinforcement effect, which generally points to the future’s fear and focuses on the surrounding people and future anxiety (Hamachek, 1978). In line with previous research, body appreciation negatively predicated body-related shame and body-related guilt (Avalos et al., 2005). Body appreciation as a positive body attitude distinguishes from body-related shame and body-related guilt, indicating body appreciation is converse to body-related shame and body-related guilt (Tangney et al., 1996; Stoeber et al., 2007). Therefore, in a clinical sense, excessive focus on body appreciation will make the individual compare with the surrounding environment and people and then produce a negative emotional experience of shame and guilt. As a better suggestion, we hope that individuals can take things as they are and do not pay too much attention to their body image.

Interestingly, we found that unhealthy perfectionism is negatively related to body-related shame and body-related guilt, consistent with the previous study (Fedewa et al., 2005). Only one thing needs to be emphasized: our result indicated that healthy perfectionism is positively related to body-related shame and body-related guilt, which is different from previous. Logically, the term perfectionism is associated with a feeling of doing things perfectly. The essence of perfectionism is not the pursuit of excellence but conditional self-acceptance (Greenspon, 2000). We can stay mentally healthy in many ways and be high achievers while still striving for perfection.

Overall, findings support the role of body appreciation mediating perfectionism and body-related shame and body-related guilt. Through body appreciation, individuals with unhealthy perfectionist tendencies may reduce their body-related shame and body-related guilt, providing a new perspective for reducing shame and guilt about the body. It is not easy to fundamentally change the perfectionist characteristics of individuals who are unhealthy perfectionists. Try instilling a body appreciation attitude into unhealthy perfectionists. Then internalizing body appreciation reduces negative body evaluations and shame and guilt, suggesting new ways for unhealthy perfectionists to embrace their bodies and respect and appreciate them.

### Limitations

The current study contributes to a better understanding of the mediating mechanism underlying the relationship between perfectionism and body-related shame and body-related guilt. However, caution should be exercised when interpreting the current findings.

First, from the perspective of gender selection, this study only studied female college students’ body-related shame and body-related guilt. Male students’ body-related shame and body-related guilt are equally significant and worthy of attention. Future research can further study male college students and conduct a comparative study on male and female college students. At the same time, we ignore the sexual orientation has a specific role in evaluating the female body.

Second, based on the future application’s perspective, body appreciation is a positive body image and can be generalized and developed. Future research can put it at all the levels of society to understand the scope of body appreciation and improve people’s sense of identity and respect for the body.

### Conclusions and Future Directions

The current study is the first study investigating the mediating role of body appreciation between different aspects of perfectionism (healthy and unhealthy) and body-related shame and body-related guilt. The results support the notion that body appreciation is a mediator between different forms of perfectionism and body-related shame and body-related guilt.

As an essential part of this study, we found that health perfectionism and body appreciation negatively mediate guilt and shame. This also implies the principle of no excess. It is natural for women to love beauty, but excessive attention will often bring bad emotional experiences. As clinical researchers, we should give appropriate advice to clients in need to help them to balance the cognitive level of the body. As one of the potential effective interventions, if we can provide good physical appreciation training in the future, it will undoubtedly help to study women’s emotional experience of the body.

## Data Availability Statement

The raw data supporting the conclusions of this article will be made available by the authors, without undue reservation.

## Ethics Statement

The studies involving human participants were reviewed and approved by the Ethics Committee of Harbin Engineering University, and written informed consent was given by all the participants prior to participation. The patients/participants provided their written informed consent to participate in this study.

## Author Contributions

TY, YR, and BD, corresponding author, contributed to the conception and design of the study and took primary responsibility for communication with the journal and editorial office during the submission process, throughout peer review, and during publication. ZL, SL, and CY organized the database, performed the statistical analysis, and wrote the first draft of the manuscript, and they are equal contributors. XL and ZY contributed to manuscript revision and read and approved the submitted version. All authors contributed to the article and approved the submitted version.

## Conflict of Interest

The authors declare that the research was conducted in the absence of any commercial or financial relationships that could be construed as a potential conflict of interest.

## Publisher’s Note

All claims expressed in this article are solely those of the authors and do not necessarily represent those of their affiliated organizations, or those of the publisher, the editors and the reviewers. Any product that may be evaluated in this article, or claim that may be made by its manufacturer, is not guaranteed or endorsed by the publisher.

## References

[ref1] Avalos L. Tylka T. L. Wood-Barcalow N. (2005). The body appreciation scale: development and psychometric evaluation. Body Image 2, 285–297. 10.1016/j.bodyim.2005.06.002, PMID: 18089195

[ref2] Baron R. M. Kenny D. A. (1986). The moderator mediator variable distinction in social psychological-research—conceptual, strategic, and statistical considerations. J. Pers. Soc. Psychol. 51, 1173–1182. 10.1037/0022-3514.51.6.1173, PMID: 3806354

[ref3] Bessenoff G. R. Snow D. (2006). Absorbing society's influence: body image self-discrepancy and internalized shame. Sex Roles 54, 727–731. 10.1007/s11199-006-9038-7

[ref4] Boone L. Soenens B. Braet C. Goossens L. (2010). An empirical typology of perfectionism in early-to-mid adolescents and its relation with eating disorder symptoms. Behav. Res. Ther. 48, 686–691. 10.1016/j.brat.2010.03.022, PMID: 20430369

[ref5] Brislin R. W. (1970). Back-translation for cross-cultural research. J. Cross-Cult. Psychol. 1, 185–216. 10.1177/135910457000100301

[ref001] Brunet J. Pila E. Solomon-Krakus S. Sabiston C. M. O’Loughlin J. (2019). Self-esteem moderates the associations between body-related self-conscious emotions and depressive symptoms. J. Health Psychol. 24, 833–843. 28810377 10.1177/1359105316683786

[ref6] Burns D. D. (1980). The perfectionist’s script for self-defeat. Psychol. Today 14, 34–52.

[ref8] Cash T. F. Iii E. A. D. (1997). The nature and extent of body-image disturbances in anorexia nervosa and bulimia nervosa: a meta-analysis. Int. J. Eat. Disord. 22, 107–126. 10.1002/(SICI)1098-108X(199709)22:2<S107::AID-EAT1>3.0.CO;2-J 9261648

[ref9] Cash T. F. Theriault J. Annis N. M. (2004). Body image in an interpersonal context: adult attachment, fear of intimacy, and social anxiety. J. Soc. Clin. Psychol. 23, 89–103. 10.1521/jscp.23.1.89.26987

[ref10] Cella S. Iannaccone M. Cotrufo P. (2020). Does body-related shame mediate the relationship between parental bonding, self-esteem, maladaptive perfectionism, body mass index and eating disorders? A structural equation model. Eat Weight Disord. 25, 667–678. 10.1007/s40519-019-00670-3 30859466

[ref11] Dalley S. E. Bron G. G. Hagl I. F. A. Heseding F. Hoppe S. Wit L. (2020). Bulimic symptoms in a sample of college women: disentangling the roles of body size, body-related shame and negative urgency. Eat Weight Disord. 25, 1357–1364. 10.1007/s40519-019-00771-z, PMID: 31555972 PMC7508931

[ref12] Enns M. W. Cox B. J. (2002). The Nature and Assessment of Perfectionism: A Critical Analysis. Washington DC: American Psychological Association, 33–62.

[ref13] Fedewa B. A. Burns L. R. Gomez A. A. (2005). Positive and negative perfectionism and the shame/guilt distinction: adaptive and maladaptive characteristics. Personal. Individ. Differ. 38, 1609–1619. 10.1016/j.paid.2004.09.026

[ref14] Fei Z. Xu Z. (2006). Reliability and validity of the frost multidimensional perfectionism questionnaire. Chin. J. Clin. Psych. 14, 560–563. 10.3969/j.issn.1005-1252.2005.05.001

[ref15] Ferreira C. Trindade I. A. Ornelas L. (2015). Exploring drive for thinness as a perfectionistic strategy to escape from shame experiences. Span. J. Psychol. 18:E29. 10.1017/sjp.2015.27 25990841

[ref16] Fiske L. Fallon E. A. Blissmer B. Redding C. A. (2014). Prevalence of body dissatisfaction among United States adults: review and recommendations for future research. Eat. Behav. 15, 357–365. 10.1016/j.eatbeh.2014.04.010, PMID: 25064281

[ref17] Flett G. L. Hewitt P. L. (2002). “Perfectionism and maladjustment: an overview of theoretical, definitional, and treatment issues,” in Perfectionism: Theory, Research, and Treatment. eds. Flett G. L. Hewitt P. L. (Washington, DC, US: American Psychological Association), 5–31.

[ref18] Franco-Paredes K. Mancilla-Díaz J. M. Vázquez-Arévalo R. López-Aguilar X. Álvarez-Rayón G. (2005). Perfectionism and eating disorders: a review of the literature. Eur. Eat. Disord. Rev. 13, 61–70. 10.1002/erv.605 17960773

[ref19] Frederick D. A. Forbes G. B. Grigorian K. E. Jarcho J. M. (2007). The UCLA body project I: gender and ethnic differences in self-objectification and body satisfaction among 2,206 undergraduates. Sex Roles 57, 317–327. 10.1007/s11199-007-9251-z

[ref20] Frederick D. A. Sandhu G. Morse P. J. Swami V. (2016). Correlates of appearance and weight satisfaction in a U.S. national sample: personality, attachment style, television viewing, self-esteem, and life satisfaction. Body Image 17, 191–203. 10.1016/j.bodyim.2016.04.001, PMID: 27172812

[ref21] Fredrickson B. L. Roberts T. A. (1997). Objectification theory—toward understanding women's lived experiences and mental health risks. Psychol. Women Q. 21, 173–206. 10.1111/j.1471-6402.1997.tb00108.x

[ref22] Frost R. O. Marten P. Lahart C. Rosenblate R. (1990). The dimensions of perfectionism. Cogn. Ther. Res. 14, 449–468. 10.1007/BF01172967

[ref23] Giancarlo D. Lysaker P. H. Teresa C. Roberto P. Nicola M. Ilaria R.. (2015). Perfectionism and personality disorders as predictors of symptoms and interpersonal problems. Am. J. Psychother. 69, 317–330. 10.1176/appi.psychotherapy.2015.69.3.317, PMID: 26414312

[ref24] Gol H. C. Rostami A. M. Goudarzi M. (2013). Prediction of marital satisfaction based on perfectionism. Procedia Soc. Behav. Sci. 89, 567–571. 10.1016/j.sbspro.2013.08.896

[ref25] Greenspon T. S. (2000). “Healthy perfectionism” is an oxymoron! Reflections on the psychology of perfectionism and the sociology of science. J. Second. Gift. Educ. 11, 197–208. 10.4219/jsge-2000-631

[ref26] Hamachek D. E. (1978). Psychodynamics of normal and neurotic perfectionism. Psychology 15, 27–33.

[ref27] Hayes A. F. (2013). Introduction to Mediation, Moderation, and Conditional Process Analysis: A Regression-Based Approach. New York, NY: The Guilford Publications.

[ref28] Hedman E. Strom P. Stunkel A. Mortberg E. (2013). Shame and guilt in social anxiety disorder: effects of cognitive behavior therapy and association with social anxiety and depressive symptoms. PLoS One 8:e61713. 10.1371/journal.pone.0061713, PMID: 23620782 PMC3631156

[ref29] Hewitt P. L. Flett G. L. (1991). Perfectionism in the self and social contexts—conceptualization, assessment, and association with psychopathology. J. Pers. Soc. Psychol. 60, 456–470. 10.1037/0022-3514.60.3.456, PMID: 2027080

[ref30] Hewitt P. L. Flett G. L. Sherry S. B. Habke M. Parkin M. Lam R. W.. (2003). The interpersonal expression of perfection: porfectionistic self-presentation and psychological distress. J. Pers. Soc. Psychol. 84, 1303–1325. 10.1037/0022-3514.84.6.1303, PMID: 12793591

[ref31] Hoek H. W. van Hoeken D. (2003). Review of the prevalence and incidence of eating disorders. Int. J. Eat. Disord. 34, 383–396. 10.1002/eat.10222, PMID: 14566926

[ref32] Hongfei Y. (2007). The test of the chinese version of frostm multi-dimensional perfectionism scale. Chin. Ment. Health J. 21, 97–100.

[ref33] Iannantuono A. C. Tylka T. L. (2012). Interpersonal and intrapersonal links to body appreciation in college women: an exploratory model. Body Image 9, 227–235. 10.1016/j.bodyim.2012.01.004, PMID: 22401976

[ref35] Lewis H. B. (1971). Shame and guilt in neurosis. Psychoanal. Rev. 58, 419–438. PMID: 5150685

[ref36] Marta-Simões J. Ferreira C. (2016). Seeking a perfect body look: feeding the pathogenic impact of shame? Eat Weight Disord. 21, 477–485. 10.1007/s40519-015-0240-x 26590601

[ref37] Mensinger J. L. Tylka T. L. Calamari M. E. (2018). Mechanisms underlying weight status and healthcare avoidance in women: a study of weight stigma, body-related shame and guilt, and healthcare stress. Body Image 25, 139–147. 10.1016/j.bodyim.2018.03.001, PMID: 29574257

[ref39] Pacht A. R. (1984). Reflections on perfection. Am. Psychol. 39, 386–390. 10.1037/0003-066X.39.4.386

[ref40] Peterson J. S. (2003). An argument for proactive attention to affective concerns of gifted adolescents. J. Educ. Gift. 14, 62–70. 10.4219/jsge-2003-419

[ref41] Pidgeon A. M. Appleby L. (2014). Investigating the role of dispositional mindfulness as a protective factor or boy image dissatisfaction among women. Curr. Res. Soc. Psychol. 5, 96–103. 10.3844/crpsp.2014.96.103

[ref42] Piotrowski K. (2019). Perfectionism and identity processes in two domains: mediational roles of worry, rumination, indecisiveness, shame, and guilt. Front. Psychol. 10:1864. 10.3389/fpsyg.2019.01864, PMID: 31507476 PMC6716423

[ref46] Slevec J. H. Tiggemann M. (2011). Predictors of body dissatisfaction and disordered eating in middle-aged women. Clin. Psychol. Rev. 31, 515–524. 10.1016/j.cpr.2010.12.002, PMID: 21239098

[ref47] Stice E. Shaw H. E. (2002). Role of body dissatisfaction in the onset and maintenance of eating pathology: a synthesis of research findings. J. Psychosom. Res. 53, 985–993. 10.1016/S0022-3999(02)00488-9, PMID: 12445588

[ref48] Stoeber J. Harris R. A. Moon P. S. (2007). Perfectionism and the experience of pride, shame, and guilt: comparing healthy perfectionists, unhealthy perfectionists, and non-perfectionists. Personal. Individ. Differ. 43, 131–141. 10.1016/j.paid.2006.11.012

[ref49] Stoeber J. Otto K. (2006). Positive conceptions of perfectionism: approaches, evidence, challenges. Personal. Soc. Psychol. Rev. 10, 295–319. 10.1207/s15327957pspr1004_2, PMID: 17201590

[ref50] Stumpf H. Parker W. D. (2000). A hierarchical structural analysis of perfectionism and its relation to other personality characteristics. Pers. Individ. Differ. 28, 837–852. 10.1016/S0191-8869(99)00141-5

[ref51] Subirana I. Sanz H. Vila J. (2020). Building Bivariate Tables: The compareGroups Package for R [Article]. J. Stat. Softw. 57, 518–524. 10.18637/jss.v057.i12

[ref52] Swami V. Ng S. K. Barron D. (2016). Translation and psychometric evaluation of a standard Chinese version of the body appreciation scale-2. Body Image 18, 23–26. 10.1016/j.bodyim.2016.04.005, PMID: 27236474

[ref53] Tangney J. P. (1992). Situational determinants of shame and guilt in young adulthood. Personal. Soc. Psychol. Bull. 18, 199–206. 10.1177/0146167292182011

[ref54] Tangney J. P. (1998). “How does guilt differ from shame?” in Guilt & children. ed. Bybee J. (San Diego, CA: Academic Press), 1–17.

[ref55] Tangney J. P. Miller R. S. Flicker L. Barlow D. H. (1996). Are shame, guilt, and embarrassment distinct emotions? J. Pers. Soc. Psychol. 70, 1256–1269. 10.1037/0022-3514.70.6.1256, PMID: 8667166

[ref57] Tylka T. L. Wood-Barcalow N. L. (2015). The body appreciation scale-2: item refinement and psychometric evaluation. Body Image 12, 53–67. 10.1016/j.bodyim.2014.09.006, PMID: 25462882

[ref58] Webb J. B. Wood-Barcalow N. L. Tylka T. L. (2015). Assessing positive body image: contemporary approaches and future directions. Body Image 14, 130–145. 10.1016/j.bodyim.2015.03.010, PMID: 25910972

[ref59] Wei T. Simko V. (2017). R package “Corrplot”: visualization of a correlation matrix (version 0.90,2020.06.30). Available at: https://cran.r-project.org/web/packages/corrplot/

[ref60] Weingarden H. Renshaw K. D. Tangney J. P. Wilhelm S. (2016). Development and validation of the body-focused shame and guilt scale. J. Obsessive Compuls. Relat. Disord. 8, 9–20. 10.1016/j.jocrd.2015.11.001, PMID: 26640760 PMC4665112

[ref61] White J. Halliwell E. (2010). Examination of a sociocultural model of excessive exercise among male and female adolescents. Body Image 7, 227–233. 10.1016/j.bodyim.2010.02.002, PMID: 20206589

[ref62] Wood-Barcalow N. L. Tylka T. L. Augustus-Horvath C. L. (2010). “But I Like my body”: positive body image characteristics and a holistic model for young-adult women. Body Image 7, 106–116. 10.1016/j.bodyim.2010.01.001, PMID: 20153990

[ref63] Yager Z. Gray T. Curry C. McLean S. A. (2017). Body dissatisfaction, excessive exercise, and weight change strategies used by first-year undergraduate students: comparing health and physical education and other education students. J. Eat. Disord. 5:10. 10.1186/s40337-016-0133-z, PMID: 28392918 PMC5376693

